# Early treatment with tolvaptan improves diuretic response in acute heart failure with renal dysfunction

**DOI:** 10.1007/s00392-017-1122-1

**Published:** 2017-05-24

**Authors:** Yuya Matsue, Jozine M. ter Maaten, Makoto Suzuki, Sho Torii, Satoshi Yamaguchi, Seiji Fukamizu, Yuichi Ono, Hiroyuki Fujii, Takeshi Kitai, Toshihiko Nishioka, Kaoru Sugi, Yuko Onishi, Makoto Noda, Nobuyuki Kagiyama, Yasuhiro Satoh, Kazuki Yoshida, Peter van der Meer, Kevin Damman, Adriaan A. Voors, Steven R. Goldsmith

**Affiliations:** 1 Department of Cardiology, University of Groningen, University Medical Center Groningen, Groningen, The Netherlands; 2 Department of Cardiology, Kameda Medical Center, 929, Higashi-Cho, Kamogawa, Chiba Japan; 3 Department of Cardiology, Tokai University School of Medicine, Kanagawa, Japan; 4 Department of Cardiology, Tomishiro Central Hospital, Okinawa, Japan; 5 Department of Cardiology, Tokyo Metropolitan Hiroo Hospital, Tokyo, Japan; 6 Department of Cardiology, Ome Municipal General Hospital, Tokyo, Japan; 7 Department of Cardiology, Yokohama Minami Kyosai Hospital, Kanagawa, Japan; 8 Department of Cardiovascular Medicine, Kobe City Medical Center General Hospital, Kobe, Japan; 9 Department of Cardiology, Saitama Medical Center, Saitama Medical University, Kawagoe, Japan; 10 Division of Cardiovascular Medicine, Toho University Ohashi Medical Center, Tokyo, Japan; 11 Department of Cardiology, Hiratsuka Kyosai Hospital, Kanagawa, Japan; 12 Department of Cardiology, Tokyo Yamate Medical Center, Tokyo, Japan; 13 Department of Cardiology, The Sakakibara Heart Institute of Okayama, Okayama, Japan; 14 Department of Cardiology, National Disaster Medical Center, Tokyo, Japan; 15 Department of Epidemiology, Harvard T. H. Chan School of Public Health, Boston, MA USA; 16 Division of Cardiology, Hennepin County Medical Center and University of Minnesota, Minneapolis, MN USA

**Keywords:** Acute heart failure, Diuretics, Worsening renal function, Dyspnea relief

## Abstract

**Background:**

Poor response to diuretics is associated with worse prognosis in patients with acute heart failure (AHF). We hypothesized that treatment with tolvaptan improves diuretic response in patients with AHF.

**Methods:**

We performed a secondary analysis of the AQUAMARINE open-label randomized study in which a total of 217 AHF patients with renal impairment (eGFR < 60 mL/min/1.73 m^2^) were randomized to either tolvaptan or conventional treatment. We evaluated diuretic response to 40 mg furosemide or its equivalent based on two different parameters: change in body weight and net fluid loss within 48 h.

**Results:**

The mean time from patient presentation to randomization was 2.9 h. Patients with a better diuretic response showed greater relief of dyspnea and less worsening of renal function. Tolvaptan patients showed a significantly better diuretic response measured by diuretic response based both body weight [−1.16 (IQR −3.00 to −0.57) kg/40 mg vs. −0.51 (IQR −1.13 to −0.20) kg/40 mg; *P* < 0.001] and net fluid loss [2125.0 (IQR 1370.0–3856.3) mL/40 mg vs. 1296.3 (IQR 725.2–1726.5) mL/40 mg; *P* < 0.001]. Higher diastolic blood pressure and use of tolvaptan were independent predictors of a better diuretic response.

**Conclusions:**

Better diuretic response was associated with greater dyspnea relief and less WRF. Early treatment with tolvaptan significantly improved diuretic response in AHF patients with renal dysfunction.

**Electronic supplementary material:**

The online version of this article (doi:10.1007/s00392-017-1122-1) contains supplementary material, which is available to authorized users.

## Introduction

Volume overload and subsequent congestion are the primary causes and treatment targets for acute heart failure (AHF) [1, 2]. Diuretics have, therefore, been the mainstay of treatment of patients with AHF [3]. Recent studies, however, have suggested that there are patients with AHF who may be refractory to conventional diuretic therapy [4, 5]. This poor diuretic response is a strong and independent predictor of unfavorable prognosis [6], and no therapy has yet been proven to benefit patients with a poor diuretic response.

Tolvaptan is an oral, non-peptide, selective vasopressin-2 receptor antagonist, and prevents the activation of the aquaporin system and impairs the ability of the kidney to reabsorb water; as a result, free water excretion is increased. In the Efficacy of Vasopressin Antagonism in Heart Failure Outcome Study With Tolvaptan (EVEREST) trial, tolvaptan showed a favorable short-term effect but neutral long-term effect in AHF patients [7]. However, in this trial AHF patients were enrolled relatively late after presentation as a consequence of inclusion criteria (<48 h from hospitalization). Recent AHF studies have showed “time to treatment” is a factor associated with drug efficacy and patient prognosis [8, 9] and the latest European Society of Cardiology heart failure guideline emphasizes the importance of treating AHF patients as quickly as possible [10]. Therefore, treatment with tolvaptan in the very early phase worth evaluating. Moreover, no study has evaluated diuretic response in Asian AHF patients. In the AQUAMARINE study (a randomized study evaluated efficacy of tolvaptan in patients with AHF and renal dysfunction), all patients were randomized within 6 h from hospitalization. Consequently, median time from first presentation to randomization was 2.1 h. In this study, we aimed to evaluate the effect of early treatment with tolvaptan on diuretic response in AHF patients with concomitant renal dysfunction.

## Methods

### Study population

This is a retrospective secondary analysis of the AQUAMARINE study. The study design and primary results of AQUAMARINE have been described elsewhere [11, 12]. In brief, 217 patients with AHF and renal dysfunction (estimated glomerular filtration rate, 15–60 mL/min/1.73 m^2^) were randomized within 6 h from hospitalization into two groups, either tolvaptan treatment or conventional treatment, to evaluate the efficacy of early treatment with tolvaptan. Fifty-three patients (48.6%) in the tolvaptan group received tolvaptan for more than 2 days, and no patient who was initially allocated to conventional group crossed over to tolvaptan during the first 48 h. The protocol of the study was approved by the ethics committees of all participating centers, and written informed consent was obtained from all the participants. This trial was registered at UMIN-CTR (Unique identifier: UMIN000007109).

### Data collection

In the AQUAMARINE study, data regarding blood pressure, heart rate, and improvement in dyspnea from baseline and blood samples were collected at 6, 12, 24, and 48 h from enrollment. Dyspnea was assessed according to the patient-reported seven-point Likert scale. Within 48 h, the amount of furosemide-equivalent loop diuretics, change in body weight from baseline, and urine output were noted down. Worsening renal function was defined as an increase of ≥0.3 mg/dL in the serum creatinine from the baseline at various pre-specified time points (6, 12, 24, and 48 h from randomization). The incidence of the combined endpoints for all-cause mortality and re-hospitalization for heart failure within 90 days was also evaluated.

### Diuretic response

We defined diuretic response as the change in body weight (kg) from baseline to 48 h per 40 mg intravenous furosemide administration. Oral furosemide was converted to half the dose of intravenous furosemide. The doses of oral loop diuretics that were considered equivalent to 40 mg intravenous furosemide were 10 mg torasemide and 60 mg azosemide [13, 14]. We also performed analyses using net fluid loss within 48 h as a measure of diuretic response. Diuretic response was measured according to body weight change in 189 cases after excluding 28 cases due to missing data on the total diuretic dose (*n* = 3) and body weight change (*n* = 25). Data on diuretic response based on net fluid loss were achieved in 171 cases and missing in 46 cases due to unavailability of information on water intake in 45 cases and on furosemide dose in 3 cases.

### Statistical analysis

Data were expressed as mean ± standard deviation for normally distributed variables and as median with interquartile range (IQR) for non-normally distributed data. Categorical data were expressed as numbers and percentages. The relationships between baseline characteristics, outcomes and tertiles of diuretic response were compared by one-way analysis of variance, Kruskal–Wallis test, or χ^2^ test, as appropriate. Correlation analysis was performed using Spearman’s rho. When necessary, variables were transformed for further analyses. Stepwise multiple linear regression analysis was performed using backward elimination method after including all variables with P values below 0.10 in the univariate analysis. Statistical analyses were performed using R version 3.1.2.

## Results

In the AQUAMARINE study, 220 patients were originally enrolled, of which 217 were analyzed because one patient in the tolvaptan group and one patient in the conventional group withdrew their consent and data were missing for one patient in the tolvaptan group. The baseline characteristics of randomized patients were shown elsewhere [12]. The median age of the patients was 75 years (interquartile range [IQR], 68–81 years), and 64.9% was male. The median left ventricular ejection fraction was 44.5%, and 82 (37.8%) patients had a left ventricular ejection fraction ≥50%. Mean baseline eGFR was 40.5 mL/min/1.73 m^2^, and 57 (26.3%) patients had an eGFR < 30 mL/min/1.73 m^2^. Time from first-medical record input to randomization was obtained in 210 (96.8%) patients, and it was 2.9 h in mean, and 2.1 h in median. Time from patient appearance to randomization and the place they appear was shown in Supplemental Figure 1.

During the first 48 h from study enrollment, the median administered amount of furosemide-equivalent diuretic dose was 100 mg (IQR, 62.5–160 mg), median total body weight change was −2.30 kg (IQR −3.50 to −1.18 kg), and median net fluid loss was 3973.0 mL (IQR 2566.3–5410.0 mL). The median values for the measures of diuretic response were −0.83 (IQR −1.50 to −0.40) kg/40 mg body weight and 1582.8 (IQR 895–2478.3) mL/40 mg net fluid loss. The baseline characteristics of the study population according to diuretic response tertiles are shown in Table 1. Using baseline characteristics, poor diuretic response based on change in body weight, was associated with less edematous status, less history of hypertension, and more hyponatremia. These associations were retained for diuretic response based on net fluid loss. In correlation analysis, change in body weight and net fluid loss showed a statistically significant, but relatively weak correlation (Spearman’s rho = − 0.47, *P* < 0.001) (Supplemental Figure 2).

**Table 1:** Baseline characteristics and relationship among tertiles of diuretic response

Diuretic response (per 40 mg furosemide-equivalent) [min–max]	Diuretic response with body weight changes (kg/40 mg furosemide)				Diuretic response with net-fluid loss (mL/40 mg furosemide)			
	Tertile1 (best)	Tertile2	Tertile3 (worst)	*P* value	Tertile1 (best)	Tertile2	Tertile3 (worst)	*P* value
	(*N* = 66)	(*N* = 61)	(*N* = 62)		(*N* = 57)	(*N* = 57)	(*N* = 57)	
	−2.42	−0.80	−0.21		4427.5	2046.9	1009.3	
	[−10.6 to −1.20]	[−1.20 to −0.50]	[−0.48 to 4.00]		[2875.0–21520.0]	[1634.0–2843.3]	[98.2–1577.1]	
Age	72 ± 8	72 ± 12	74 ± 9	0.456	73 ± 8	73 ± 9	71 ± 11	0.498
Male (%)	46 (69.7)	42 (68.9)	37 (59.7)	0.421	37 (64.9)	41 (71.9)	35 (61.4)	0.482
Body weight at baseline	63.0 (56.0–69.7)	60.0 (51.0–68.7)	60.1 (49.9–69.5)	0.409	63.0 (55.9–68.3)	61.8 (55.0–71.4)	64.1 (52.6–70.3)	0.975
SBP (mmHg)	141 ± 26	139 ± 31	137 ± 25	0.767	144 ± 24	140 ± 29	135 ± 26	0.158
DBP (mmHg)	83 ± 19	78 ± 22	77 ± 19	0.188	84 ± 18	78 ± 18	78 ± 18	0.113
HR (bpm)	96 ± 30	89 ± 25	90 ± 24	0.213	94 ± 28	92 ± 24	91 ± 25	0.808
Edema at baseline^a^ (%)				0.074				0.376
None	6 (9.1)	8 (13.1)	11 (18.0)		5 (8.8)	8 (14.0)	7 (12.5)	
Mild	15 (22.7)	25 (41.0)	23 (37.7)		15 (26.3)	13 (22.8)	21 (37.5)	
Moderate	23 (34.8)	16 (26.2)	18 (29.5)		19 (33.3)	24 (42.1)	18 (32.1)	
Severe	22 (33.3)	12 (19.7)	9 (14.8)		18 (31.6)	12 (21.1)	10 (17.9)	
Edema moderate/severe at baseline (%)	45 (68.2)	28 (45.9)	27 (44.3)	0.01	37 (64.9)	36 (63.2)	28 (50)	0.212
Orthopnea at baseline (%)	48 (72.7)	35 (57.4)	37 (59.7)	0.149	39 (68.4)	39 (68.4)	38 (66.7)	0.974
Pulmonary congestion at baseline (%)	64 (97.0)	53 (86.9)	60 (96.8)	0.031	55 (96.5)	50 (87.7)	55 (96.5)	0.088
NYHA III/IV (%)	48 (72.7)	36 (59.0)	39 (62.9)	0.245	42 (73.7)	39 (68.4)	42 (73.7)	0.771
Ischemic etiology (%)	15 (22.7)	13 (21.3)	20 (32.3)	0.313	16 (28.1)	13 (22.8)	15 (26.3)	0.807
LVEF (%)	43.3 ± 17.5	48.7 ± 16.1	47.7 ± 17.3	0.161	46.2 ± 19.1	43.7 ± 17.1	47.8 ± 15.9	0.443
Medical history (%)								
HF admission	26 (39.4)	26 (42.6)	32 (51.6)	0.358	28 (49.1)	21 (36.8)	28 (49.1)	0.314
Hypertension	56 (84.8)	41 (67.2)	50 (80.6)	0.046	52 (91.2)	35 (62.5)	43 (75.4)	0.001
Diabetes	29 (43.9)	23 (37.7)	30 (48.4)	0.486	27 (47.4)	24 (42.1)	24 (42.1)	0.808
Dyslipidemia	33 (50.0)	19 (31.1)	29 (46.8)	0.116	28 (49.1)	24 (42.1)	19 (33.3)	0.288
Atrial fibrillation	38 (57.6)	31 (50.8)	32 (51.6)	0.596	30 (52.6)	31 (54.4)	32 (56.1)	0.705
Smoking (current or ex)	43 (65.2)	38 (63.3)	33 (55.0)	0.467	34 (60.7)	38 (67.9)	31 (58.5)	0.57
Drugs at admission (%)								
ACE-I	6 (9.1)	7 (11.5)	1 (1.6)	0.092	6 (10.5)	4 (7.0)	4 (7.0)	0.733
ARB	19 (28.8)	16 (26.2)	27 (43.5)	0.085	18 (31.6)	13 (22.8)	20 (35.1)	0.336
Beta blocker	25 (37.9)	21 (34.4)	25 (40.3)	0.795	21 (36.8)	19 (33.3)	27 (47.4)	0.279
Aldosterone antagonist	14 (21.2)	9 (14.8)	18 (29.0)	0.157	11 (19.3)	11 (19.3)	8 (14.0)	0.695
Digoxin	4 (6.1)	3 (4.9)	4 (6.5)	0.931	4 (7.0)	3 (5.3)	3 (5.3)	0.899
Diuretics	27 (40.9)	23 (37.7)	28 (45.2)	0.701	20 (35.1)	25 (43.9)	24 (42.1)	0.6
Furosemide equivalent dose among users (mg)	40 (5–80)	40 (10–200)	40 (10–120)	0.356	40 (10–80)	20 (10–120)	40 (5–200)	0.575
Tolvaptan treatment (%)	47 (71.2)	33 (54.1)	17 (27.4)	<0.001	46 (80.7)	26 (45.6)	19 (33.3)	<0.001
IV therapy within 48 h (%)								
Carperitide	25 (37.9)	23 (37.7)	19 (30.6)	0.628	25 (43.9)	24 (42.1)	12 (21.1)	0.018
Dopamine	1 (1.5)	0 (0.0)	2 (3.2)	0.359	0 (0.0)	2 (3.5)	0 (0.0)	0.132
Dobutamine	5 (7.6)	5 (8.2)	9 (14.5)	0.359	3 (5.3)	7 (12.3)	8 (14.0)	0.271
Nitrate	16 (24.2)	10 (16.4)	9 (14.5)	0.321	16 (28.1)	8 (14.0)	11 (19.3)	0.172
Vasodilator	17 (25.8)	10 (16.4)	11 (17.7)	0.359	16 (28.1)	9 (15.8)	13 (22.8)	0.286
Heparin	34 (51.5)	19 (31.1)	25 (40.3)	0.065	32 (56.1)	17 (29.8)	24 (42.1)	0.018
Lab data at baseline								
Creatinine	1.5 ± 0.6	1.3 ± 0.5	1.5 ± 0.5	0.148	1.4 ± 0.5	1.4 ± 0.5	1.5 ± 0.7	0.728
eGFR	38.3 ± 13.9	43.5 ± 12.2	38.9 ± 13.4	0.056	39.1 ± 13.6	42.0 ± 13.2	40.2 ± 14.2	0.503
BUN	28 (20–35)	26 (19–35)	28 (20–37)	0.891	24 (19–32)	28 (18–35)	28 (22–35)	0.466
Na	141 ± 4	141 ± 3	139 ± 4	0.017	141 ± 4	140 ± 4	139 ± 5	0.149
K	4.4 ± 0.6	4.2 ± 0.5	4.4 ± 0.7	0.288	4.4 ± 0.6	4.3 ± 0.5	4.3 ± 0.8	0.583
BNP	939.3 (544.4–1477.6)	866.9 (492.0–1554.1)	750.2 (393.5–1463.6)	0.373	897.9 (572.1–1622.0)	726.3 (491.0–1094.6)	1009.1 (393.5–1716.2)	0.189
Dose of diuretics use within 48 h (mg)	60 (23–100)	100 (80–140)	140 (100–200)	<0.001	60 (20–80)	100 (80–140)	160 (110–220)	<0.001
Urine volume within 48 h (mL)	6584.1 ± 3559.0	6192.2 ± 2399.2	4500.3 ± 1679.1	<0.001	7319.9 ± 3599.0	5974.9 ± 1877.2	4399.8 ± 1906.5	<0.001
Water intake within 48 h (mL)	1867.3 ± 1434.1	1685.3 ± 986.4	1446.0 ± 933.4	0.183	1902.5 ± 1456.2	1497.4 ± 739.4	1488.3 ± 1054.2	0.082
Net fluid loss within 48 h (mL)	4747.9 ± 2839.2	4419.8 ± 1999.0	3344.2 ± 1594.1	0.006	5417.3 ± 2768.2	4477.5 ± 1718.9	2911.5 ± 1675.1	<0.001
Body weight changes within 48 h (kg)	−3.8 (−5.3 to −2.6)	−2.4 (−2.9 to −1.6)	−0.9 (−1.5 to 0.0)	<0.001	−3.0 (−4.7 to −2.0)	−2.4 (−3.3 to −1.3)	−1.4 (−2.5 to −0.8)	<0.001

^a^Data on edema at baseline was missing in one patient

To identify predictors of diuretic response, univariable (Supplemental Table 1) and multivariable linear regression analysis (Table 2) for both parameters were performed. The only independent predictors of a good diuretic response for both criteria were tolvaptan use and a higher diastolic blood pressure. There was no interaction between baseline diuretics and tolvaptan on diuretic response for both BW definition (*P* value for interaction = 0.816) and net fluid loss definition (*P* value for interaction = 0.642). Likewise, no significant interaction was observed between baseline sodium level, renal function, and impact of tolvaptan treatment on diuretic response (all *P* value for interaction >0.20). For both diuretic response definitions, no interaction was found on the effect of tolvaptan on diuretic response between patients who were treated with and without carperitide (*P* for interaction = 0.137 with body weight definition and 0.707 with net fluid loss definition).

**Table 2:** Multivariable linear regression analysis of diuretic response

Variable	Standardized beta	*t*	*P* value
Diuretic response with body weight changes (kg/40 mg furosemide)			
Adjusted *R* ^2^ = 0.214			
Tolvaptan treatment	−0.339	−5.246	<0.001
Heparin IV	−0.241	−3.707	<0.001
DBP	−0.149	−2.279	0.024
Edema (moderate/severe)	−0.137	−2.088	0.011
Diuretic response with net fluid loss (mL/40 mg furosemide)			
Adjusted *R* ^2^ = 0.176			
Tolvaptan treatment	0.387	5.495	<0.001
DBP	0.199	2.854	0.005

Patients with a poor diuretic response were less likely to have an improvement in dyspnea relief within 48 h from randomization, as defined by moderate or marked improvement from baseline according to the seven-point Likert scale (Table 3). A poor diuretic response was also significantly associated with more WRF (Table 3). A worse diuretic response was not associated with an increased risk of pre-specified prognostic endpoints within 90 days.

**Table 3:** Outcomes of the tertiles of diuretic response

Diuretic response (per 40 mg furosemide-equivalent) [min–max]	Diuretic response with body weight changes (kg/40 mg furosemide)				Diuretic response with Net-fluid loss (mL/40 mg furosemide)			
	Tertile 1 (good)	Tertile 2	Tertile 3	*P* value	Tertile 1 (good)	Tertile 2	Tertile 3	*P* value
	(*N* = 66)	(*N* = 61)	(*N* = 62)		(*N* = 57)	(*N* = 57)	(*N* = 57)	
	−2.42	−0.8	−0.21		4427.5	2046.9	1009.3	
	[−10.6 to −1.20]	[−1.20 to −0.50]	[−0.48 to 4.00]		[2875.0–21520.0]	[1634.0–2843.3]	[98.2–1577.1]	
Dyspnea relief (moderately or markedly)								
6 h	15 (22.7)	10 (16.4)	8 (12.9)	0.331	14 (24.6)	8 (14.0)	8 (14.0)	0.223
12 h	25 (39.1)	20 (32.8)	10 (16.4)	0.017	24 (42.9)	18 (31.6)	11 (19.3)	0.026
24 h	35 (53.0)	31 (51.7)	12 (19.4)	<0.001	33 (57.9)	29 (50.9)	12 (21.1)	<0.001
48 h	52 (80.0)	44 (72.1)	21 (34.4)	<0.001	47 (83.9)	44 (78.6)	18 (31.6)	<0.001
WRF (Cre increase ≥0.3 mg/dL from baseline) (%)	11 (16.7)	13 (21.3)	22 (35.5)	0.037	8 (14.0)	13 (22.8)	22 (38.6)	0.009
Length of hospital stay (Days)	13.9 (8.4–18.7)	13.2 (8.5–19.4)	18.4 (10.3–27.2)	0.101	13.5 (8.9–17.4)	13.4 (10.0–20.6)	18.4 (11.2–24.4)	0.088
Prognosis within 90 days (%)								
Death	1 (1.5)	2 (3.3)	4 (6.5)	0.335	1 (1.8)	3 (5.3)	3 (5.3)	0.551
Combined of death or HF readmission	7 (10.8)	9 (14.8)	9 (14.5)	0.759	4 (7.0)	9 (15.8)	9 (15.8)	0.271

*Cre* creatinine, *HF* heart failure, *WRF* worsening renal function

**Fig. 1:**
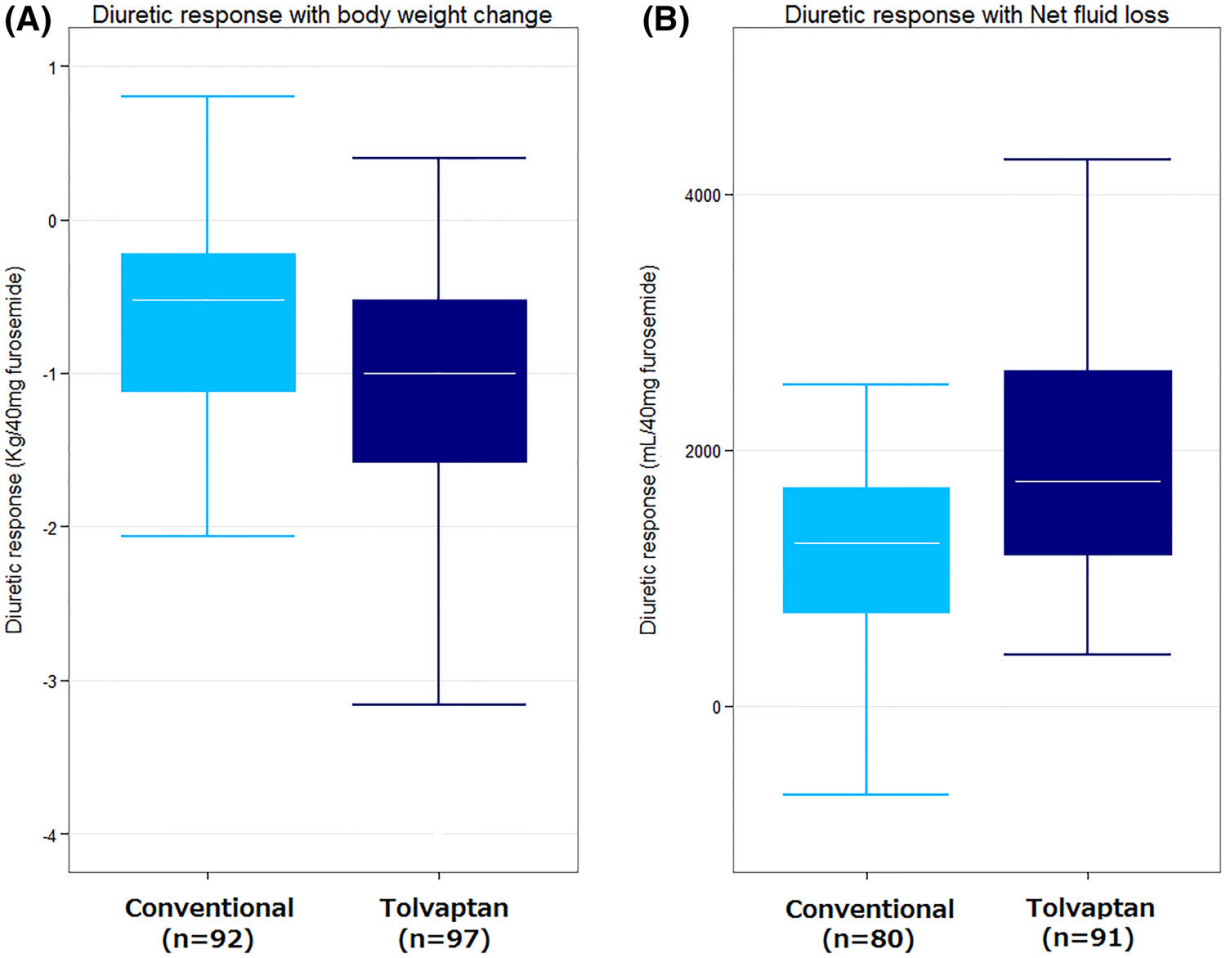
Diuretic response in patients with acute heart failure according to treatment with tolvaptan. Measurements compared were **a** change in body weight and **b** net fluid loss

Figure 1 shows the diuretic response according to randomization group, i.e., with and without tolvaptan treatment. Compared to patients who were not treated with tolvaptan, those who were treated with tolvaptan showed a significantly better diuretic response based on assessment by both body weight change [−1.16 (IQR −3.00 to −0.57) kg/40 mg vs. −0.51 (IQR −1.13 to −0.20) kg/40 mg; *P* < 0.001] and net fluid loss [2125.0 (IQR 1370.0–3856.3) mL/40 mg vs. 1296.3 (IQR 725.2–1726.5) mL/40 mg; *P* < 0.001].

## Discussion

In patients with AHF and renal dysfunction, very early treatment with tolvaptan was independently associated with better diuretic response. AHF patients with poor diuretic response had less dyspnea relief and more frequently experienced worsening renal function.

### Diuretic response in AHF

In spite of the lack of a universal definition, poor response to diuretic therapy has been shown to be one of the most powerful prognostic predictors in patients with heart failure [4, 13, 15, 16]. Initial studies used diuretic dose to define diuretic response, i.e., patients with persistent heart failure despite treatment with a certain dose of diuretics were defined to have a “poor diuretic response” [15, 17]. However, this definition used only amount of diuretics and hereby obviously ignored response to the diuretics and, therefore, assumed equal effectiveness. Recently, a novel definition of diuretic response based on urine/body weight response to a certain amount of diuretics was proposed [6]. In all studies that evaluated its prognostic potential, diuretic response consistently showed significant prognostic ability in patients with AHF when this modified definition was used [4, 5, 18, 19].

There has been no consensus on the parameter used to measure diuretic response to 40 mg furosemide or its equivalent, although recent studies have used either change in body weight, net fluid loss, or total urinary output. In the present study, we showed relatively poor correlation between the two measures of diuretic response. This result was in line with that of the DOSE trial and ASCEND-HF, which demonstrated a poor agreement between net fluid loss and weight loss [5, 20]. It is clear that we need better measures of diuretic response to encompass natriuretic response, change in volume distribution, and change in hemodynamic status. However, our consistent results on the improvement of diuretic response, by two different parameters, with tolvaptan supported our hypothesis.

According to this novel definition, the median diuretic response was −0.51 kg/40 mg/48 h furosemide in the conventional group in our study. This was greater than approximately 0.4 kg/40 mg of furosemide-equivalent diuretic response in the patients of the ASCEND-HF (weight change from admission to 48 h), RELAX-AHF (weight change from day 1 to 5), and PROTECT (weight change from day 1 to 4) studies [4, 5, 18]. This better diuretic response in this AQUAMARINE cohort did not match our expectations because our study included only AHF patients with renal dysfunction on admission and earlier studies suggest that renal dysfunction predisposes to worse diuretic response [4, 5, 18]. There are several conceivable speculations for this unexpected result. First, lower doses of loop diuretic were given in AQUAMARINE, compared to other studies and the additional effect of a drug usually decreases at higher doses. Second, although baseline creatinine values were higher in the AQUAMARINE cohort than in the ASCEND-HF cohort, levels of baseline blood urea nitrogen were not substantially different between these two studies. Given that blood urea nitrogen, but not creatinine, has been suggested by previous studies as the most powerful determinant of diuretic response [4, 21], this may be one of the reasons for discrepancy in our study. Third, median time till randomization from patient arrival was 2.1 h and 41.4% of all AQUAMARINE cohort was randomized before admission at the emergency department or clinic. This is surprisingly short given that mean time from admission to randomization was 15.5 h in ASCEND-HF and 7.9 h in RELAX-AHF [22]. This means AQUAMARINE randomized AHF patients much earlier, and we could, therefore, evaluate diuretic response in the very early phase which was not possible with previous diuretic response studies in AHF cohorts. This difference in the time window might be associated with the unexpected good diuretic response in our study cohort. Finally, our results lead to hypothesis that there may be a racial difference in diuretic response. All of the studies regarding diuretic response so far predominantly enrolled Western AHF patients and little is currently known about diuretic response in Asian AHF patients. This hypothesis is supported by the observation that the amount of intravenous loop diuretics used in the acute phase was very low (around or less than 100 mg/48 h) in Japanese AHF patients compared to Western patients [12, 23]. Therefore, influence of racial and/or genetic information on diuretic response needs to be elucidated in future studies.

For both diuretic response parameters, high blood pressure was associated with a good diuretic response. These findings were in accordance with the results of previous studies. In the PROTECT, RELAX-AHF, and ASCEND-HF cohorts, low diastolic blood pressure was an independent predictor of poor diuretic response [4, 5, 18]. Interestingly, intravenous unfractionated heparin was associated with good diuretic response measured with body weight. We have no clear explanation for this finding; however, hyperkalemia is known to be a rare but possible complication of heparin therapy [24], and hypokalemia was suggested as an independent predictor of poor diuretic response in PROTECT. Moreover, there is a case report that suggests a direct effect of heparin on diuresis in patients with AHF [25]. The association between intravenous heparin and diuretic response needs to be precisely elucidated in the future studies.

Poor diuretic response was significantly associated with a high incidence of worsening renal function and low rate of improvement in dyspnea at almost all time points. These results are in line with the findings of previous studies [4, 18]; however, it should be acknowledged that the number of events were very small and this study was obviously underpowered to evaluate prognostic significance of diuretic response.

### Effect of tolvaptan on diuretic response

Although some interventions to treat AHF patients with diuretic resistance have been investigated, there has been no proven therapy to improve diuretic resistance in this high risk population. In the ROSE-AHF study, neither low-dose nesiritide nor low-dose dopamine on top of standard of therapy was associated with a greater reduction in body weight within 72 h [26]. Given that the total amount of furosemide-equivalent diuretic used within 72 h was not significantly different, neither low-dose dopamine nor low-dose nesiritide was suggested to improve diuretic response. Likewise, in ASCEND-HF, nesiritide did not improve diuretic response [5]. In RELAX-AHF, serelaxin did not show a significant improvement in diuretic response of patients with AHF despite its potentially favorable effects on prognosis [18, 27, 28]. Rolofylline, an adenosine A1-receptor antagonist, on the other hand did improve diuretic response [4]. However, its clinical use was hampered by a neutral effect on prognosis and the concern for neurological adverse events. Ultrafiltration might be a promising decongestive strategy [29]; however, it has not been studied specifically in patients with a poor diuretic response.

In the present study, we showed that very early treatment with tolvaptan could improve diuretic response in AHF patients with renal impairment. The pathophysiological background of this favorable effect of tolvaptan on diuretic response remains to be elucidated; however, it may be attributed to certain differences in the mechanisms of action between loop diuretics and tolvaptan. First, time-dependent diuretic resistance was observed with loop diuretics. In patients who have been treated with diuretics for a long time, effectiveness is blunted gradually with time [30]. Second, loop diuretics have to be bound to plasma albumin and delivered to the proximal tubules to exert their effects. Therefore, hypoalbuminemia, which is common in patients with AHF, could contribute to poor diuretic response [31, 32]. Third, active secretion of loop diuretics into the lumen via an organic acid transporter is needed for them to act [33]. This transporter could be inhibited by endogenous organic anions [34]. However, compared with furosemide, tolvaptan has a different mechanism of action, i.e., inhibiting the activation of vasopressin-2 receptor by arginine–vasopressin and subsequent insertion of aquaporin-2 channels in the collecting tubules. This might be one of the reasons for the improvement in diuretic response in renal-impaired patients with AHF after intake of tolvaptan.

Contrary to our result, recent sub-analysis from EVEREST showed a lack of significant difference in prescription rate of tolvaptan between good/bad diuretic response groups [35]. There are some differences in patient backgrounds between EVEREST and AQUAMARINE that possibly explain this discordance (e.g., racial difference, baseline renal function). However, the most conceivable explanation for this discrepancy is time to treatment. In EVEREST, time from hospitalization to dyspnea assessment (the next calendar day after the first drug administration) was more than 36 h in 47.7%, and more than 60 h in 20.2% [36]. In AQUAMARINE about 40% of all patients were randomized before admission to the hospital ward and this early capture of AHF patients may lead to short time to randomization and better diuretic response. The association between time to therapy and diuretic response in AHF patient needs to be addressed in future studies.

Tolvaptan is expected to cause aquaresis but not natriuresis. As sodium retention plays a pivotal role in pathophysiology of AHF, aquaresis may have a different impact on prognosis from natriuresis in AHF patients. Although the pathophysiological background of the association between diuretic response and prognosis has yet to be elucidated, early successful decongestion and subsequent symptom relief are plausible mechanisms. Given that several studies, including AQUAMARINE, have consistently showed urine output with tolvaptan (i.e., aquaresis) could also lead to decongestion and subsequent symptom relief, improvement of diuretic response with early treatment with tolvaptan in AHF patients potentially improves outcome. From this perspective, EVEREST might not be suitable to evaluate this hypothesis as tolvaptan was used relatively late and did not improve diuretic response. As we showed improvement in diuretic response with very early treatment with tolvaptan for the first time, future studies on early use of tolvaptan for patients with AHF having poor diuretic response are warranted.

Our study had several limitations; primarily, its open-label design, which could have influenced some subjective prognostic variables, including relief of dyspnea. This study focused on short-term responses and did not have sufficient power to detect long term differences in WRF. We could not address the association between diuretic response and prognosis because of very little number of events. As we recruited and randomized patients very early in our study, some non-AHF patients might have been included. However, all patients went through careful clinical history taking, physical examination, chest X-ray and analysis of natriuretic. Only after confirmation that patients met the criteria as stated in the protocol, they were randomized and received the study drug. In addition, we performed sensitivity analyses comparing the effects of tolvaptan in patients with a BNP between 100 and 350 pg/ml and above 350 pg/mL We found no interaction in the effect of tolvaptan on diuretic response in patients with higher versus lower BNP levels at admission (*P* value for interaction = 0.183). No standardized diuretic regimen was applied and usage of diuretics was at the discretion of the treating physician. Our findings regarding association between diuretic response and dyspnea relief should be interpreted carefully because baseline severity of dyspnea was not evaluated and difference in baseline dyspnea severity between good and poor diuretic response group might affect difference in degree of dyspnea relief.

The most powerful limitation of this study which should be acknowledged is that this is a post hoc and non-pre-specified analysis. Moreover, several analyses were performed without adjusting for multiple testing. Given these points, our study result should be interpreted as an exploratory analysis and hypothesis generating.

## Conclusions

Very early treatment with tolvaptan improved diuretic response in patients with a hospital admission for AHF. Future research focusing on the prognostic implication of improving diuretic response with early treatment with tolvaptan in patients with poor diuretic response is warranted.

## Electronic supplementary material

Below is the link to the electronic supplementary material.

Supplementary material 1 (PDF 163 kb)
