# Supplementary material for: Early treatment with tolvaptan improves diuretic response in acute heart failure with renal dysfunction
Source: Clin Res Cardiol. 2017 May 24;106(10):802–12. doi: 10.1007/s00392-017-1122-1 (PMC5613036; doi:10.1007/s00392-017-1122-1)
Supplement: Supplementary file 1 — Supplementary material 1 (PDF 163 kb) [file 392_2017_1122_MOESM1_ESM.pdf]

Supplemental Materials

Supplemental Figure 1. Place of initial presentation, randomization timing, and time from presentation to randomization in the AQUAMARINE cohort

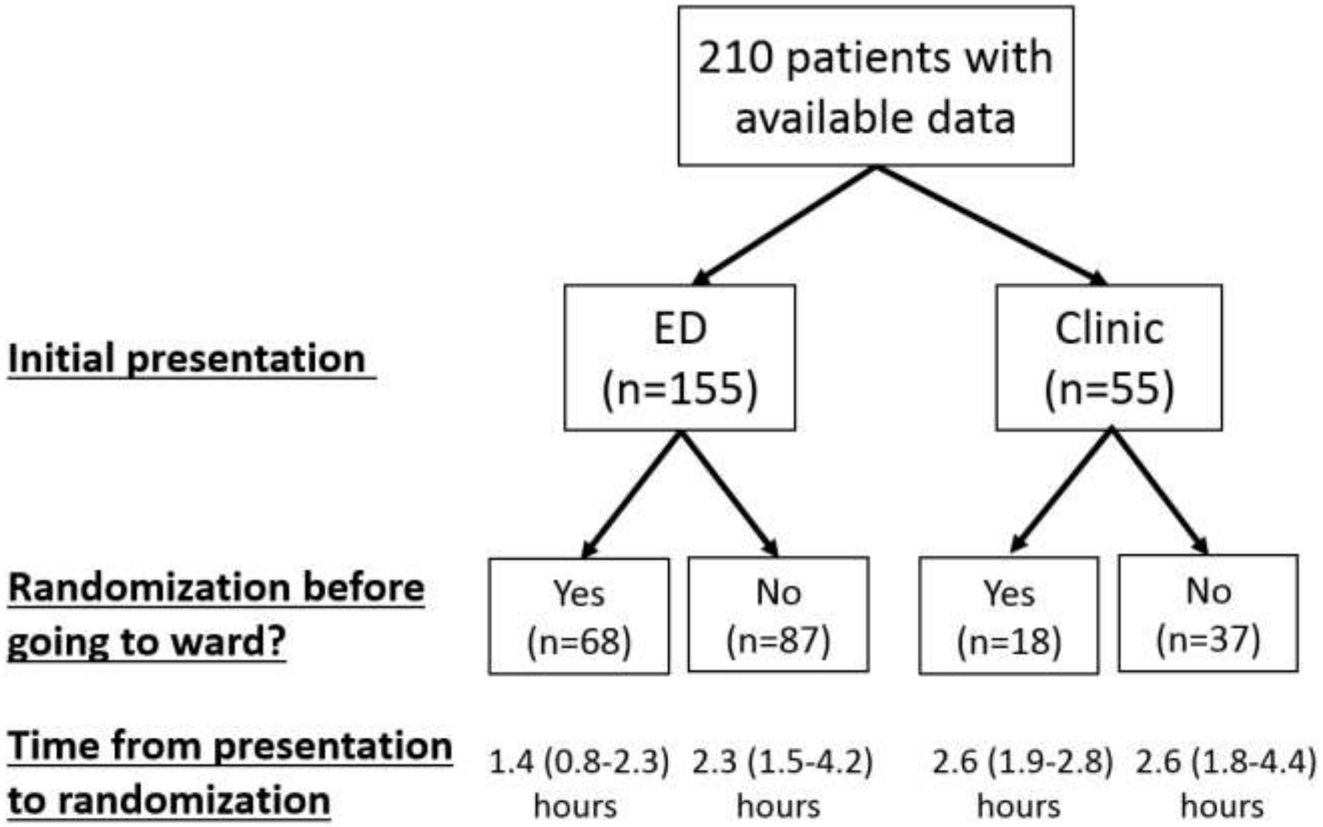

Supplemental Figure 2. Scatter plot between body weight change and net fluid loss with fit line and its 95% confidence interval (shaded area)

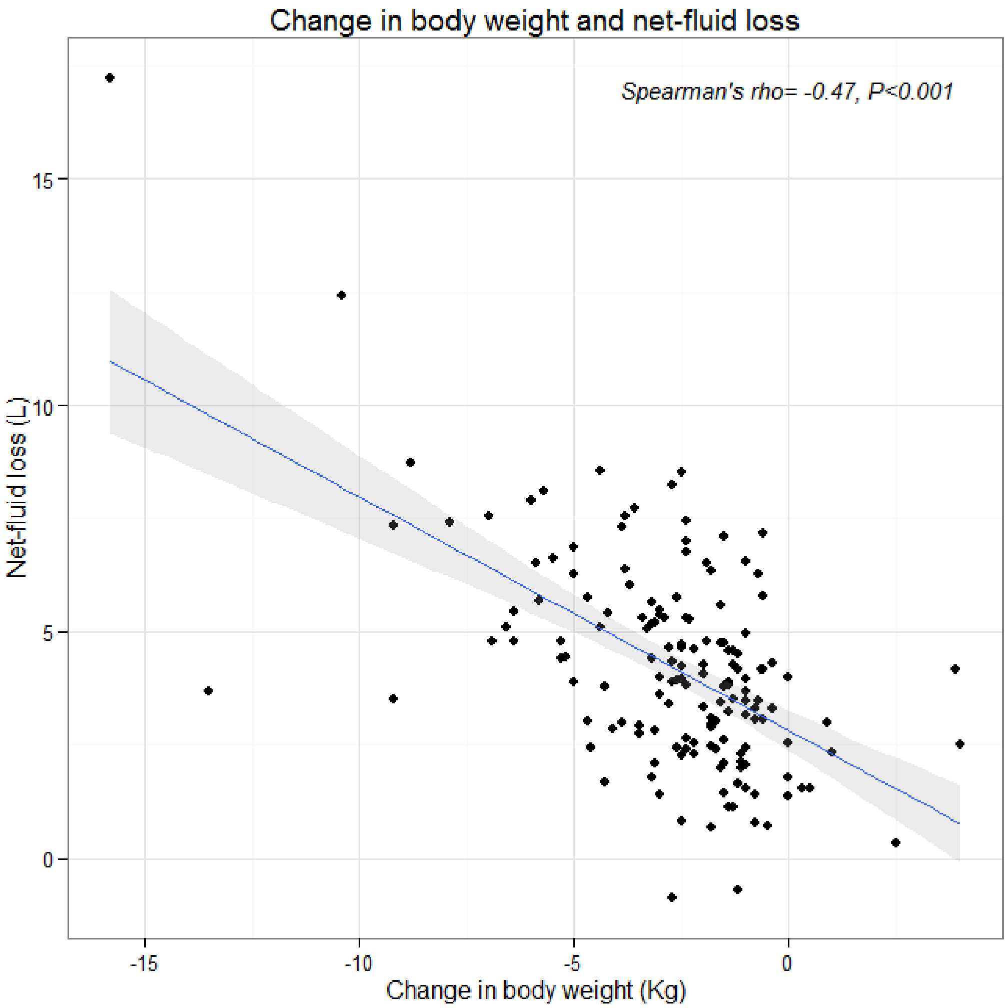

rho: Spearman's rank coefficient of correlation

Supplemental Table 1. Univariable linear regression analysis for diuretic response

Variables	Diuretic response with body weight changes (kg/40mg furosemide)			Diuretic response with Net fluid loss (mL/40mg furosemide)		
	Standardized beta	t	P value	Standardized beta	t	P value
Age (years)	0.04	0.548	0.585	-0.009	-0.121	0.904
Male (%)	-0.085	-1.169	0.244	0.003	0.045	0.964
SBP (mmHg)	-0.157	-2.177	0.031	0.156	2.047	0.042
DBP (mmHg)	-0.196	-2.73	0.007	0.152	1.982	0.049
Heart rate (bpm)	-0.12	-1.654	0.099	0.051	0.656	0.513
Left ventricular ejection fraction (%)	0.125	1.71	0.089	-0.055	-0.71	0.478
NYHA III/IV (%)	-0.046	-0.629	0.531	-0.001	-0.011	0.991
Tolvaptan treatment	-0.349	-5.092	<0.001	0.404	5.711	<0.001
Medical History (%)						
HF admission	0.079	1.086	0.279	0.016	0.2	0.841
Hypertension	-0.097	-1.333	0.184	0.1	1.299	0.196
Diabetes	-0.032	-0.433	0.666	0.001	0.009	0.993
Dyslipidemia	0.009	0.126	0.9	0.024	0.309	0.757
Atiral fibrillation	-0.025	-0.335	0.738	-0.018	-0.229	0.819
Smoking (Current or Ex)	-0.135	-1.843	0.067	0.042	0.534	0.594
Drug at admission (%)						
Furosemide equivalent dose (mg)	0.065	0.891	0.374	-0.139	-1.813	0.072
ACE	-0.042	-0.573	0.567	0.024	0.298	0.766
ARB	0.083	1.141	0.255	-0.055	-0.714	0.476
Beta blocker	-0.002	-0.025	0.98	-0.07	-0.913	0.362
Aldosterone antagonist	0.073	0.996	0.321	0.044	0.566	0.572
Digoxin	0.025	0.338	0.735	-0.018	-0.231	0.817
Time to Randomization (hour)	-0.113	-1.532	0.127	0.015	0.192	0.848
IV therapy w/i 48h (%)						
Carperitide	-0.146	-2.02	0.045	0.195	2.574	0.011
Nitrate	-0.217	-3.041	0.003	0.137	1.787	0.076
ISDN	0.045	0.611	0.542	-0.091	-1.184	0.236
Vasodilator	-0.195	-2.715	0.007	0.103	1.335	0.184
Nicorandil	0.016	0.214	0.831	-0.089	-1.149	0.252
Heparin	-0.233	-3.275	0.001	0.109	1.428	0.155

Dopamin	0.026	0.357	0.722	0.003	0.045	0.964
Dobutamin	0.117	1.619	0.107	-0.129	-1.683	0.094
Lab data						
Creatinine (mg/dL)	-0.112	-1.542	0.125	-0.031	-0.408	0.684
eGFR (mL/min/1.73m2)	0.072	0.988	0.324	-0.032	-0.417	0.677
BUN (mg/dL)	-0.007	-0.101	0.919	-0.161	-2.107	0.037
Na (mEq/L)	-0.119	-1.643	0.102	0.164	2.154	0.033
K (mEq/L)	-0.066	-0.909	0.365	0.011	0.14	0.889
BNP (pg/mL)	-0.108	-1.479	0.141	-0.045	-0.584	0.56
Body weight at admission (Kg)	-0.037	-0.505	0.615	-0.002	-0.027	0.979
Edema (moderate/severe)	-0.191	-2.647	0.009	0.177	2.317	0.022
Orthopnea	-0.105	-1.448	0.149	0.046	0.601	0.549
Pulmonary Congestion	-0.048	-0.656	0.513	0.043	0.553	0.581
Water intake	-0.069	-0.854	0.395	0.169	2.21	0.029

* Time-to-randomization was missing in 3 patients
